# Safety, efficacy and clinical generalization of the STAR protocol: a retrospective analysis

**DOI:** 10.1186/s13613-016-0125-9

**Published:** 2016-03-29

**Authors:** Kent W. Stewart, Christopher G. Pretty, Hamish Tomlinson, Felicity L. Thomas, József Homlok, Szabó Némedi Noémi, Attila Illyés, Geoffrey M. Shaw, Balázs Benyó, J. Geoffrey Chase

**Affiliations:** Department of Mechanical Engineering, Centre for Bio-Engineering, University of Canterbury, Private Bag 4800, Christchurch, 8140 New Zealand; Department of Intensive Care, Christchurch Hospital, Christchurch, New Zealand; Department of Control Engineering and Information Technology, Budapest University of Technology and Economics, Budapest, Hungary; Department of Intensive Care, Kálmán Pándy Hospital, Gyula, Hungary

**Keywords:** Glycemic control, Intensive care unit, Hyperglycemia, Hypoglycemia, Model-based, Personalized, Patient-specific, Stochastic, Targeted

## Abstract

**Background:**

The changes in metabolic pathways and metabolites due to critical illness result in a highly complex and dynamic metabolic state, making safe, effective management of hyperglycemia and hypoglycemia difficult. In addition, clinical practices can vary significantly, thus making GC protocols difficult to generalize across units.The aim of this study was to provide a retrospective analysis of the safety, performance and workload of the stochastic targeted (STAR) glycemic control (GC) protocol to demonstrate that patient-specific, safe, effective GC is possible with the STAR protocol and that it is also generalizable across/over different units and clinical practices.

**Methods:**

Retrospective analysis of STAR GC in the Christchurch Hospital Intensive Care Unit (ICU), New Zealand (267 patients), and the Gyula Hospital, Hungary (47 patients), is analyzed (2011–2015). STAR Christchurch (BG target 4.4–8.0 mmol/L) is also compared to the Specialized Relative Insulin and Nutrition Tables (SPRINT) protocol (BG target 4.4–6.1 mmol/L) implemented in the Christchurch Hospital ICU, New Zealand (292 patients, 2005–2007). Cohort mortality, effectiveness and safety of glycemic control and nutrition delivered are compared using nonparametric statistics.

**Results:**

Both STAR implementations and SPRINT resulted in over 86 % of time per episode in the blood glucose (BG) band of 4.4–8.0 mmol/L. Patients treated using STAR in Christchurch ICU spent 36.7 % less time on protocol and were fed significantly more than those treated with SPRINT (73 vs. 86 % of caloric target). The results from STAR in both Christchurch and Gyula were very similar, with the BG distributions being almost identical. STAR provided safe GC with very few patients experiencing severe hypoglycemia (BG < 2.2 mmol/L, <5 patients, 1.5 %).

**Conclusions:**

STAR outperformed its predecessor, SPRINT, by providing higher nutrition and equally safe, effective control for all the days of patient stay, while lowering the number of measurements and interventions required. The STAR protocol has the ability to deliver high performance and high safety across patient types, time, clinical practice culture (Christchurch and Gyula) and clinical resources.

## Background

Stress-induced insulin resistance, resulting in hyperglycemia, is often experienced by intensive care unit (ICU) patients [1–3] and is associated with increased morbidity and mortality [1, 2, 4]. Glycemic variability in these highly dynamic patients has also been independently related to increased mortality [5, 6]. Studies have shown that safe, effective glycemic control (GC) that modulates exogenous insulin and/or nutrition significantly reduces the number of negative effects of dysglycemia [7–9], including reductions in the rate and severity of organ failure [10] and cost of care [11, 12].

The stress response a critically ill patient experiences is highly complex, variable and dynamic [13], making safe, effective control of blood glucose (BG) difficult. This is one reason a number of studies may have failed to achieve consistent, safe and effective GC [14–20]. These negative and inconclusive outcomes could be largely due to the studies having a significant increase in the risk of hypoglycemia (17–29 % <2.2 mmol/L), which is associated with increased mortality [21–23], and can be associated with large changes in patient response to insulin over short periods [13, 24, 25], particularly in the first 48 h [26], where many hypoglycemic events occur and there is a strong association with mortality [22].

It is thus possible that previous studies failed to achieve safe consistent GC due to the GC protocols not being able to observe or identify individual patient-specific dynamics, but providing GC based on an absolute BG value. A more patient-specific approach to GC is needed to successfully manage such significant inter- and intra-patient variability [24, 27]. The ability to provide safe, effective control across patients and clinical practice is a necessary requirement before being able to assess the impact of GC on clinical outcomes.

The tablet-computer-based stochastic targeted (STAR) GC protocol provides patient-specific GC [28, 29]. STAR uses a clinically validated pharmacokinetic and pharmacodynamic model of the insulin–glucose system [30, 31] and a cohort-based model of insulin sensitivity variability [32, 33] to compute optimal patient-specific insulin and nutrition interventions that maximize control and nutrition, while maintaining a maximum 5 % risk of BG < 4.4 mmol/L [28, 29]. STAR also allows variable 1- to 3-hourly BG measurement frequency to help manage nursing workload. STAR has been the standard of care in Christchurch Hospital ICU, Christchurch, New Zealand, and in the Kálmán Pándy Hospital ICU, Gyula, Hungary, since 2011. The predecessor of STAR in Christchurch was the Specialized Relative Insulin and Nutrition Tables (SPRINT) [8, 10].

The aim of this dual-center retrospective analysis is to demonstrate that patient-specific, safe, effective GC is possible with the STAR protocol and that it is also generalizable across/over different units and clinical practices.

## Methods

### Comparisons

This dual-center retrospective analysis makes two comparisons to assess.

1. Performance and safety: STAR is compared to SPRINT in Christchurch to provide a comparison between patient protocols in the same unit, and demonstrate equivalent or better performance and safety of STAR to a successful protocol [8].
2. Generalizability: STAR Christchurch is compared to STAR Gyula to test generalizability of safety and performance over significantly different clinical practice cultures and approaches.

The metrics compared are:

- Performance: Percentage of time in BG band (4.4–8.0 mmol/L) [34].
- Safety: Number of severe hypoglycemic cases (BG < 2.2 mmol/L) [21].
- Safety: Number of moderate hypoglycemic cases (BG < 4.0 mmol/L) [22].

### Patients and protocols

#### Cohorts

This study compares clinical data from three cohorts:

1. Patients treated using STAR in Christchurch Hospital ICU, Christchurch, New Zealand, from June 2011 to May 2015.
2. Patients treated using STAR in Kálmán Pándy Hospital ICU, Gyula, Hungary, from December 2011 to May 2015.
3. Patients treated using SPRINT in Christchurch Hospital ICU, Christchurch, NZ, from July 2005 to May 2007.

The following patients in these three cohorts were excluded: those who spent less than 10 h on protocol and were fed on average greater than 120 % of their SCCM/ACCP caloric target [35]. Patients who spent less than 10 h on protocol were excluded as they were considered to not have a significant amount of time on protocol to fairly assess the performance or be clinically affected by good GC. Patients who were fed greater than 120 % of their calorific target were excluded as this is well outside the recommendations of STAR and SPRINT, as well as well-accepted clinical guidelines. The number of patient episodes excluded due to each of these filtering criteria is given in Table 1.

**Table 1:** Episode filtering statistics

Number of episodes (%)	SPRINT Christchurch	STAR Christchurch	STAR Gyula
Initial number	487	625	68
Different GC target to protocol	0 (0.0 %)	225 (36.0 %)	11 (16.2 %)
Episode length <10 h	58 (11.9 %)	49 (7.8 %)	6 (8.8 %)
Fed over 120 % target	74 (15.2 %)	15 (2.4 %)	4 (5.9 %)
Remaining for analysis	355 (72.9 %)	336 (53.8 %)	47 (69.1 %)

Patients on the STAR protocol who did not target the 4.4–8.0 mmol/L BG band were also excluded. Thus, all STAR patients compared used the STAR framework in the same manner with respect to BG and nutrition targets. It should be noted that all SPRINT patients targeted the 4.0–6.1 mmol/L BG band as the SPRINT protocol was not flexible to different targets.

Demographic data for these cohorts are presented in Tables 2 and 3, aligned with the two main comparisons. Some data are unavailable for the STAR Gyula cohort in Table 3 due to differences in the typical data collected. The missing data do not impact the assessment of generalizability of the safety and performance of STAR.

**Table 2:** Patient demographics for the STAR and SPRINT Christchurch cohorts

Cohort characteristics	SPRINT Christchurch	STAR Christchurch	*P* value
Total patients	292	267	
Age	63 [48:73]	65 [55:72]	0.28
Percent male	62.7	65.5	0.48
Length of ICU stay	6.2 [2.7:13.0]	5.7 [2.5:13.4]	0.70
% Operative	38.7	34.8	0.38
APACHE II score	19.0 [15.0:24.5]	21.0 [16.0:25]	<0.05
APACHE II RoD (%)	29.0 [16.0:51.0]	33.0 [15.0:53.0]	0.41
ICU mortality (%)	18.2	24.3	0.08
Hospital mortality (%)	26.0	30.0	0.35
Hospital SMR	0.76	0.86	–
Mortality on GC protocol (%)	5.5	6.4	0.72

Data are presented as median [inter-quartile range (IQR)] where appropriate

**Table 3:** Patient demographics for the STAR Christchurch and Gyula cohorts

Cohort characteristics	STAR Gyula	STAR Christchurch	*P* value
Total patients	47	267	
Age	66 [58:71]	65 [55:72]	0.72
Percent male	61.7	65.5	0.62
Length of ICU stay	14 [8.0:20.5]	5.7 [2.5:13.4]	<0.05
APACHE II score	32.0 [28.0:36.0]	21.0 [16.0:25]	<0.05
ICU Mortality (%)	38.3	24.3	0.05
Mortality on GC protocol (%)	0	6.4	0.09

Data are presented as median [inter-quartile range (IQR)] where appropriate

#### Ethics, consent and permissions

The Upper South Regional Ethics Committee, New Zealand, granted approval for the retrospective audit, analysis and publication of the Christchurch patient data. According to the local ethical codes in Hungary, the study of the Gyula cohort was considered a clinical data audit and only required depersonalization of data without the need for individual patient consent to analyze or publish the anonymized data.

#### Clinical practice and implementation: Christchurch Hospital ICU, New Zealand

STAR has been the standard of care in the Christchurch Hospital ICU since June 2011. This facility is a mixed medical, tertiary affiliated ICU. Starting criteria for STAR in Christchurch is two successive BG measurements over 8 mmol/L within a 4-h period. IV insulin is delivered in hourly bolus form, with added background infusions of up to 3U/h when insulin requirements are high and sustained [28, 29]. Blood for BG measurement was typically taken directly from an arterial line and measured using an Arkray Super-Glucocard™ II glucometer (Arkray, Minnesota, USA) (2011–2012) or a Roche Accu-Chek Inform II (F. Hoffmann-La Roche Ltd, Basel, Switzerland) (2012–2015).

SPRINT, the predecessor to STAR, used the same entry criteria as STAR and used the same insulin delivery procedures [8, 10]. Blood for BG measurement was also typically taken directly from an arterial line and measured using an Arkray Super-Glucocard™ II glucometer (Arkray, Minnesota, USA). Both SPRINT and STAR rely on closely related models of the glucose–insulin system [30, 36]. SPRINT was a paper-based protocol that was developed using the model to optimize recommended insulin and nutrition delivery based on measured BG and previous interventions [8]. However, in use, SPRINT could not explicitly calculate insulin sensitivity, or forward-predict the outcomes of interventions. In contrast, STAR implements the glucose–insulin model on tablet computer and therefore identifies insulin sensitivity, allowing forward prediction of interventions to optimize treatments, directly manage the risk of hypoglycemia and personalize care [28].

For both protocols in Christchurch Hospital ICU, patients received a similar feed type. SPRINT patients are typically fed enterally with either Glucerna™ 1 Cal. (34.3 % carbohydrate, 16.7 % protein, 14.4 g/L fiber, Abbott Labs, Illinois, USA) or Diabetic Resource (36 % carbohydrate, 24 % protein, 12 g/L fiber, Nestle Health Science, Epalinges, Switzerland). Similarly, STAR patients are typically fed Glucerna™ Select (31 % carbohydrate, 20 % protein, 21.1 g/L fiber, Abbott Labs, Illinois, USA). Note all carbohydrate concentrations exclude indigestible fiber. All the formulas used are within 1–4 % of total carbohydrate and protein content and thus provide very similar nutrition content for the patients over all of the years. For both protocols, parenteral nutrition is used occasionally to supplement enteral nutrition when necessary.

For both protocols, the same ACCP guidelines are used to determine the patients daily calorific goal intake of 25 kcal/kg/day [35] and enteral nutrition is advised between 30 and 100 % of this calorific goal [8, 28], although fixed nutrition rates and rates up to 120 % of calorific goal were included in this study. The main difference between the SPRINT and STAR feeding regime was SPRINT modulated feeding in steps up to ±10 % and effectively targeted 60–70 % of calorific goal [8], whereas STAR modulated feeding in steps up to ±30 % and targeted 100 % of calorific goal [28].

#### Clinical practice and implementation: Gyula Hospital ICU, Hungary

Kálmán Pándy County Hospital (Gyula, Hungary), which is also a mixed ICU, has been using STAR in the medical ICU since December 2011. This ICU is markedly different from Christchurch in terms of clinical GC practices. IV insulin is delivered via continuous infusion, and local nutrition guidelines specify aggressive early parenteral nutrition to supplement enteral nutrition to a similar goal feed rate of 25 kcal/kg/day. Patients are transitioned from parenteral to enteral nutrition as their stay progresses, and STAR modulates both rates to obtain total delivery values between 30 and 100 % of the daily goal.

Starting criteria for STAR in Gyula are also two successive BG measurements over 8 mmol/L within a 4-h period, but are subject to the clinician’s choice, depending on expected length of stay and severity of illness of the patient. This difference in patient selection is given in Table 3, with the Gyula cohort having much higher Apache II scores and ICU length of stay. BG is measured using the E77 Elektronika Dcont Optimum or Dcont Personal Glucometers (E77, Budapest, Hungary) with blood taken directly from an arterial line. It should be noted that only one STAR tablet is available for use in the Kálmán Pándy County Hospital, thus limiting the patient numbers and increasing the severity of illness of these patients, as it was typically used for the most ill patients.

### Patients and episodes of GC

As a single patient may be treated by a protocol on several distinct occasions separated by significant breaks, a distinction is made between a *patient* and an *episode* of GC. A *patient* is considered to be a person with the same ICU admission number, and an *episode* is considered to be a period of contiguous treatment by GC. Therefore, there can be multiple episodes per patient.

An episode was defined as a period of GC (10 h or more) in which there are no breaks in BG measurements longer than 5 h. If a gap in data exceeded 5 h, it was considered that GC had been stopped and restarted. Considering the maximum measurement interval is 3 h, this choice accounts for reasonable variance in measurement intervals.

### Analysis and statistics

The mortality on GC was calculated by working out the number of patients that died while on the GC protocol or within 5 h of the GC protocol ending. This statistic is used to identify patients for whom GC might have impacted their ICU mortality.

Blood glucose performance statistics are presented in median and inter-quartile range of individual patients mean and standard deviations of blood glucose as per Finfer et al. [36]. All hypoglycemia and other rare occurrences were manually verified. Due to irregular sampling intervals, patient episode BG data were also analyzed after linear interpolation at 60-min intervals. The BG values at 60-min intervals (either clinical or interpolated) are presented in median and inter-quartile range of individual episodes mean and standard deviations of BG, as above.

The mean hourly nutrition rates of glucose reported in Table 5 are nutrition rates excluding the hours patients were not fed, as occasionally patients could not be fed due to clinical reasons irrespective of the GC protocol. The standardized mortality ratio (SMR) calculated in Table 2 was calculated using the APACHE II Risk of Death.

Nonparametric statistics are used exclusively for all the comparative tests due to the typically skewed distributions of BG, insulin dose and other data. *P* values were computed using the Mann–Whitney rank-sum test for all continuous data and the Chi-squared test for categorical data. *P* values <0.05 are considered statistically significant.

## Results

### STAR versus SPRINT Christchurch

The cohort demographics in Table 2 show the SPRINT and STAR Christchurch cohorts have no significant difference in gender, age, operative status or ICU length of stay. However, the STAR cohort had higher APACHE II scores and ICU mortality rates than the SPRINT cohort.

Table 5 presents the per-patient and per-episode GC safety and performance of STAR and SPRINT in Christchurch. These results show that both STAR and SPRINT protocols resulted in over 86 % time in the BG band of 4.4–8.0 mmol/L per episode (86.6 and 93.0 %, respectively), while maintaining safe control of severe hypoglycemia (BG < 2.2 mmol/L, 1.5 vs. 0.3 % of patients, respectively). SPRINT’s lower and tighter BG target range (4.4–6.1 mmol/L), resulted in an increased incidence of moderate hypoglycemia (BG < 4.0 mmol/L, 62.0 vs. 26.3 % of patients for SPRINT and STAR, respectively) compared with STAR.

Table 4 presents the cohort results of GC safety and performance of STAR and SPRINT in Christchurch. In targeting this 4.4–8.0 mmol/L range using model-predictive methods to personalize treatment, STAR reduced clinical workload in the same ICU (13.6 measurements per day per patient compared to 15.8, *P* < 0.05, Table 4) and increased nutrition delivery per episode compared with SPRINT (achieving 86 % of ACCP calorific goal feed compared to 73 %, *P* < 0.05, Table 5), when allowed to feed. It did so while maintaining consistent GC for a more critically ill and thus potentially more metabolically variable cohort [13].

**Table 4:** Cohort glycemic control results for the STAR and SPRINT cohorts in Christchurch and Gyula Hospital ICU

	SPRINT Chch	STAR Chch	STAR Gyula	*P* value	
				SPRINT Chch, STAR Chch	STAR Gyula, STAR Chch
Number patients	292	267	47	–	–
Number episodes	355	336	47	–	–
Total hours	40931	22948	6244	–	–
Number of BG measurements	26530	12363	3050	–	–
Median (IQR) days on protocol	3.0 [1.3:6.3]	1.9 [0.9:3.5]	3.9 [1.9:6.9]	<0.05	<0.05
Median (IQR) measures/day per patient	15.8 [14.1:18.0]	13.6 [11.5:16.2]	11.7 [10.9–13.3]	<0.05	<0.05
*Glycemic performance—cohort raw data*					
BG mean	5.8	7.0	6.8	–	–
BG SD	1.3	1.3	1.3	–	–
BG median [IQR]	5.7 [5.0–6.6]	6.8 [6.0–7.9]	6.7 [5.8–7.8]	<0.05	<0.05
*Glycemic performance—cohort hourly interpolated*					
BG mean	5.7	6.7	6.6	–	–
BG SD	1.2	1.2	1.2	–	–
BG median [IQR]	5.6 [5.0–6.4]	6.6 [6.0–7.4]	6.5 [5.9–7.2]	<0.05	<0.05
% time >10.0 mmol/l	1.5	4.4	3.0	–	–
% time 4–6.1 mmol/l (SPRINT target)	71.4	43.9	46.5	–	–
% time 4.4–8.0 mmol/l (STAR target)	87.2	82.6	85.7	–	–
% time <4.4 mmol/l	7.4	1.4	1.9	–	–
% time <4.0 mmol/l	2.5	0.6	0.9	–	–
% time <2.22 mmol/l	0.002	0.004	0	–	–

Data are presented as median [inter-quartile range (IQR)] where appropriate

**Table 5:** Per-patient and per-episode glycemic control results for the STAR and SPRINT cohorts in Christchurch and Gyula Hospital ICU

	SPRINT Chch	STAR Chch	STAR Gyula	*P* value	
				SPRINT Chch, STAR Chch	STAR Gyula, STAR Chch
Number patients	292	267	47	–	–
Number episodes	355	336	47	–	–
*Glycemic performance—per-patient raw data*					
Median [IQR] BG mean	5.9 [5.5–6.3]	7.0 [6.6–7.6]	6.9 [6.6–7.4]	<0.05	0.60
Median [IQR] BG SD	1.2 [1.0–1.6]	1.5 [1.2–2.1]	1.6 [1.3–1.9]	<0.05	0.50
Median [IQR] BG median	5.7 [5.3–6.1]	6.7 [6.3–7.3]	6.6 [6.3–7.1]	<0.05	0.28
# (%) Patients <4.0 mmol/l	181 (62.0 %)	70 (26.3 %)	25 (53.2 %)	<0.05	<0.05
# (%) Patients <2.22 mmol/l	1 (0.3 %)	4 (1.5 %)	2 (4.3 %)	0.20	0.22
*Glycemic performance—per-episode hourly interpolated*					
Median [IQR] BG mean	5.8 [5.4–6.2]	6.7 [6.4–7.3]	6.7 [6.5–7.1]	<0.05	0.60
Median [IQR] BG SD	1.1 [0.8–1.5]	1.2 [0.9–1.7]	1.3 [1.04–1.5]	<0.05	0.23
Median [IQR] BG median	5.6 [5.2–6.0]	6.5 [6.2–7.0]	6.5 [6.3–6.7]	<0.05	0.61
% time >10.0 mmol/l	0.0 [0.0–1.6]	1.6 [0.0–6.5]	2.4 [0.7–5.4]	<0.05	0.16
% time 4–6.1 mmol/l (SPRINT target)	65.6 [52.4–77.9]	29.8 [12.4–45.9]	33.3 [21.5–40.9]	<0.05	0.49
% time 4.4–8.0 mmol/l (STAR target)	93.0 [85.0––97.5]	86.6 [75.0–94.1]	87.1 [79.3–91.1]	<0.05	0.81
% time <4.4 mmol/l	7.3 [2.1–16.1]	0.0 [0.0–1.8]	0.9 [0.0–2.8]	<0.05	<0.05
% time <4.0 mmol/l	1.4 [0.0–5.71]	0.0 [0.0–0.0]	0.0 [0.0–1.8]	<0.05	<0.05
% time <2.22 mmol/l	0.0 [0.0–0.0]	0.0 [0.0–0.0]	0.0 [0.0–0.0]	0.97	0.71
*Intervention performance—per episode (excluding not fed)*					
Median [IQR] mean insulin (U/h)	2.2 [0.0–2.8]	2.7 [1.9–3.5]	3.2 [2.4–4.6]	<0.05	<0.05
Total hours not fed (%)	16430 (40.1 %)	2305 (10.0 %)	0 (0.0 %)	–	–
Median [IQR] mean goal feed (%)	73 [52–86]	86 [64–97]	80 [74–88]	<0.05	0.28
Median[IQR] mean total glucose(g/h)	4.2 [3.1–5.4]	5.1 [4.0–6.2]	7.4 [6.2–8.9]	<0.05	<0.05
Median [IQR] mean enteral glucose (g/h)	4.1 [3.0–5.3]	4.5 [2.6–5.6]	3.04 [1.48–5.40]	0.74	<0.05
Median [IQR] mean parenteral glucose (g/h)	0.0 [0.0–0.0]	0.0 [0.0–1.1]	4.05 [2.84–5.69]	<0.05	<0.05

Data are presented as median [inter-quartile range (IQR)] where appropriate

### STAR Christchurch versus STAR Gyula

The cohort demographics in Table 3 show the STAR Christchurch and Gyula cohorts have no significant difference in gender or age. However, the STAR Gyula had much higher APACHE II scores (32 vs. 21, *P* < 0.05), ICU length of stay (14 vs. 5.7 days, *P* < 0.05) and ICU mortality rates (38.3 % vs. 24.3, *P* = 0.05) than the STAR Christchurch cohort.

Despite a significant increase in the severity of illness, STAR demonstrated consistent effective GC performance over the Gyula cohort. Table 5 shows that both Christchurch and Gyula STAR cohorts achieved over 86 % of time in its target range (4.4–8.0 mmol/l) per episode (86.6 and 87.1 %, respectively, *P* = 0.81), while maintaining very safe control of hypoglycemia per episode, <4.0 mmol/L (0.0 and 0.9 % of time, respectively, *P* < 0.05).

**Fig. 1:**
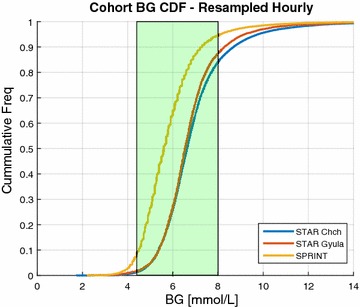
BG CDF—resampled hourly. Cumulative density plots comparing the STAR Christchurch, STAR Gyula, SPRINT Christchurch cohorts hourly resampled BG. The target band 4.4–8.0 mmol/L is shown in the *green bar*

Figure 1 shows the STAR BG distributions in these two ICUs are almost identical although *P* < 0.05 due to very large number of measurements. The per-patient BG mean and standard deviation were also very similar for both of the cohorts (*P* = 0.6 and 0.5, respectively). Thus, this evidence suggests that STAR is able to deliver consistent GC to different cohorts with significantly different clinical practices and illness severity.

Table 4 presents the cohort results of GC safety and performance of STAR in Christchurch and Gyula. Compared with the Christchurch, the Gyula clinical staff chose to use longer intervention intervals (11.7 vs. 13.6 measurements per day, *P* < 0.05) and feed a significantly higher amount of glucose per episode (5.1 vs. 7.4 g/h, *P* < 0.05, Table 5), largely due to their higher carbohydrate parenteral feed regime, thus demanding higher insulin dosing per episode (2.7 vs. 3.2 U/h, *P* < 0.05, Table 5) and amplifying the effects of insulin sensitivity variations [24]. This ultimately resulted in the Gyula cohort having a higher occurrence of moderate hypoglycemia (26.3 vs. 53.2 % of patients BG < 4.0 mmol/L, *P* < 0.05).

## Discussion

### Cohort Demographics

Table 2 shows that there has been a shift in severity of illness in the Christchurch ICU over the past 7 years, with the patients on STAR being more critically ill on average than those on SPRINT. This phenomenon is not wholly unexpected. Increasing economic and demographic stress worldwide has placed greater demand on limited bed spaces. In highly occupied units like Christchurch (2.2 beds/1000 people), admission may be limited to the more critically ill [37].

The apparent increase in ICU mortality of patients on STAR compared with SPRINT could be due to the ICU experiencing a reduction in pressure to transfer patients not expected to survive to the ward. The hospital SMR reported in Table 2 shows an increase between the SPRINT and STAR Christchurch cohorts (0.76 vs. 0.86, respectively). This suggests that the APACHE II risk of death scores is slightly more representative of the cohort’s illness in the STAR cohort compared with the SPRINT cohort. The significant difference in the severity of illness between the STAR Gyula cohort and the Christchurch cohorts is largely due to the different entrance criteria, as only one STAR tablet computer is available and STAR is thus reserved for the more critically ill patients, as clinically selected.

### STAR versus SPRINT

Compared with SPRINT, both STAR cohorts had slightly lower time in the 4.4–8.0 mmol/L band per episode (86.6 and 87.1 % vs. 93.0 %). SPRINT targeted a lower and tighter range (4.4–6.1 mmol/L), resulting in an increased incidence of moderate hypoglycemia (BG < 4.0 mmol/L, 62.0 vs. 26.3 % and 53.2 % of patients) compared with STAR. However, recent studies have shown that time in the essentially same band, 3.9–7.8 mmol/L, is associated with improved outcomes [34], supporting this slightly higher upper target limit of 8.0 mmol/L.

Flexibility to patient-specific requirements is a critical aspect of the STAR model-based protocol as the tablet application allows nursing staff to enter any information that may change the patient’s insulin or nutrition requirements. This approach allows STAR to adapt to patient-specific needs and provide appropriate recommendations that take into account all necessary considerations. Conversely, STAR achieved 96.8 % compliance from nursing staff (unchanged interventions) for nutrition delivery and 99.5 % for insulin interventions.

SPRINT was deliberately designed to target nutrition to 60–70 % or lower for control, which reduced insulin requirements and thus risk of hypoglycemia [31, 38]. SPRINT had a maximum 2-h measurement rate for the same reasons of risk mitigation which increased workload relative to STAR’s 3-h maximum. In addition, the tighter SPRINT target (4.4–6.1 mmol/L) is also part of the reason for this increased nurse workload and lower feeding on SPRINT. For STAR, the use of stochastic risk models and virtual patient design in silico enabled the lower workload and play a role in the higher nutrition possible, although a wider overlapping target band also enables some of this increased nutrition

Both STAR and SPRINT Christchurch have the same starting and stopping criteria. However, patients on STAR Christchurch required insulin therapy for 36.7 % fewer days than SPRINT (*P* < 0.05, Table 4). Differences that could account for this significant reduction in length of treatment for a more critically ill cohort include:

1. Significantly reduced incidence of moderate hypoglycemia (BG < 4.0 mmol/L) with STAR.
2. Slightly higher median BG with STAR, with similar BG in the 4.4–8.0 mmol/L range.
3. Increased mortality on the STAR protocol.
4. Increased amount of carbohydrate, protein, fiber and nutrition content delivered by STAR.

The number of patients experiencing moderate hypoglycemia is not enough to account for the size of this change. A recent analysis by Krinsley et al. [34] suggests that the 3.9–7.8 mmol/L band, similar to that targeted by STAR, is beneficial. Despite the lower median BG for SPRINT, the time in the 4.4–8.0 mmol/L band is very similar for both protocols. ICU and hospital mortality were higher for STAR due to changes in the illness severity and demographic factors, as given in Table 2. However, the percentage mortality while on the respective GC protocol is very similar (5.5 vs. 6.4 %, *P* = 0.72, Table 2), suggesting that the reduction in period of GC is less likely to be attributed to the cohorts respective mortality. This outcome leaves the increased carbohydrate, protein, fiber and overall nutrition intake as a potential reason for the reduced length of GC. However, there is significant debate about the role of energy and protein delivery in critical illness [39], with some literature showing increased calorific intake levels that are still below the 100 % calorific goal have beneficial effects [40] and others showing no effect [41]. In this study, the median per-episode feed for the STAR Christchurch cohort was 86 % of calorific goal feed, shown by Heyland et al. [42] to be a region of reduced risk of death.

### STAR Christchurch versus STAR Gyula

STAR entry criteria were the same in both units based on hyperglycemia (BG > 8.0 mmol/L) or clinician choice. STAR was used for a patient through their entire stay or multiple episodes of stay in Gyula and is the standard of care for the Christchurch ICU for all GC. Hence, the study, while a retrospective analysis, is run essentially prospectively in that all patients who had STAR with the same target band were included from each unit.

One difference between Gyula and Christchurch cohorts is that with a single tablet to run STAR in Gyula, clinician’s likely chose the more ill patients they thought would benefit. This choice could likely have biased the severity of illness upwards, as well as the length of stay (Table 3). It may have similarly affected the difference in ICU mortality in Table 3, although this comparison is significantly underpowered given the low patient numbers in Gyula and could thus also be due to statistical variation. Hence, variation in these cohorts is biased by this choice, and the results in Table 3 could be due to one or a combination of these factors, making GC in this cohort easier or more difficult depending on the patients selected. However, this difference is not as relevant to showing that STAR can provide safe, effective GC results across very different patients and clinical practices.

In the STAR Christchurch cohort, four patients experienced severe hypoglycemia, and in the Gyula cohort, two patients experienced severe hypoglycemia. STAR’s model-based predictive GC forward predicts potential patient-specific behavior using the 5th and 95th percentile, choosing an intervention to optimize the placement of these stochastic bounds [28, 33]. Therefore, all of the hypoglycemic cases occurred outside of STAR’s cohort-based model-predictive bounds. Each of these patients had a 99th percentile (outlier) patient-specific model-based insulin sensitivity during their hypoglycemic episode, and the number of events suggests this is a 1-in-100 event, which broadly matches the cohort results given in Table 5.

### Limitations

A total of 19 patients were excluded from the STAR Christchurch (15) and Gyula (4) analysis as they received more than 120 % of ACCP recommended caloric target on average. In some instances, the high level of nutrition may be clinically specified, but in most cases the high level of nutrition is due to nursing staff choosing to fix enteral and/or parenteral feed on STAR.

The discrepancy in patient numbers between the two STAR cohorts, over approximately the same time period, is due to only one of STAR tablet being available in Gyula, compared with 10 in Christchurch. The number of patients in the Gyula cohort is low, and therefore, we do not have enough power (52 %, *p* test) for comparison to the Christchurch cohort in relation to raw mortality on the STAR GC protocol in Gyula, or morbidity and per-patient BG statistics (e.g., per-patient TIB, mean/median BG). Sufficiently more patients would be required for a well powered outcome study on mortality or morbidity.

It should be noted that there was patient selection bias for STAR Gyula, creating significantly different cohorts in terms of ICU length of stay and severity of illness (*P* < 0.05, Table 3). The difference in the cohorts could result in GC being more complex or simpler in the Gyula cohort relative to the Christchurch cohort due to more severely ill patients being more variable or possibly longer length of stay patients being more stable.

It is worth noting that both severe hypoglycemic events on STAR, in Christchurch, occurred at high fixed nutrition rates over 100 % of ACCP goal feed, but <120 %. High fixed nutrition rates raise a patients BG all else equal, resulting in the need for higher or maximum insulin rates to control hyperglycemia. The large amount of insulin in the patient then amplifies any small changes in the patient’s insulin sensitivity due to changes in condition, significantly reducing the ability to control BG safely and effectively [24]. Despite high fixed nutrition inputs, up to 120 % goal feed in some cases, making glycemic control more difficult, it is also an important feature of the STAR application, allowing it to be more flexible to specific clinical needs. However, in these specific cases lower (100 % or less) and/or variable nutrition would have allowed greater safety [28].

As mentioned previously, STAR and SPRINT target different BG bands (4.4–8.0 mmol/L and 4.4–6.1 mmol/L, respectively). These BG targets overlap but vary in width and level of BG, and therefore, the comparison of these two protocols in terms of percentage of time in 4.4-8.0 mmol/L BG band may be an unfair statistic considering that only one the protocols actually targeted this band. The only way to fairly compare the two protocols performance would be if they both targeted the same BG target.

To compare the STAR GC protocol to other ICU GC protocols is difficult as the cohorts need to be similar, and the same statistics need to be reported. Considering performance, safety and workload to achieve them, other studies have shown similar performance based on time in band [43–46]. STAR achieved these performance and safety results with a lower relative clinical workload over a much larger, more diverse and relatively very ill cohort. With the data available, it appears that STAR performs very well in comparison with other current ICU GC protocols with better safety and lower workload. However, a best protocol cannot be determined without the same statistics being reported and the cohorts being much more similar.

Sensor error can affect the quality of control [47], particularly in target to value protocols [48]. SPRINT was designed in silico to be robust to these errors for the Arkray Super-Glucocard™ II glucometers used (CV < 10 % [49]). STAR is more robust to this error as its integral-based identification of insulin sensitivity in the model filters the error [50], and its target to range approach and the outlying changes in insulin sensitivity in the stochastic model used to guide control are more robust to these errors. In addition, Christchurch Hospital ICU changed to the more reliable Accu-Chek Infor II glucometer in 2012 (CV ≤ 3.2 % [51]). The Gyula unit used a E77 Elektronika Dcont Optimum or Dcont Personal Glucometers (CV < 4.6 %).

By using model-based predictive GC tailored to patient-specific metabolic response, through identifying their respective time variant insulin sensitivity, safe and effective GC can be offered. With the stochastic models of insulin sensitivity variability used, STAR thus directly manages inter- and intra-patient variability to improve safety. Other protocols could have failed in the past due to their inability to adjust for this inter- and intra-patient variability via model-based GC on a computer. Considering that this study is a retrospective analysis, as opposed to a randomized control trial, we cannot explicitly link the outcomes to GC. Thus, the main focus of this study is to demonstrate that safe, effective and generalizable GC is possible.

## Conclusions

This retrospective, observational study analyzed the GC safety, performance and generalizability of the STAR protocol in Christchurch, New Zealand and Gyula, Hungary. Results of STARs predecessor, SPRINT, were presented for comparison. Patients on the STAR protocol spend over 86 % of all time on protocol within the goal 4.4-8.0 mmol/L BG band, with very few occurrences of severe or moderate hypoglycemia. STAR outperformed SPRINT by providing higher nutrition and safe, effective control for all days of stay, as well as reducing time on protocol and workload.

Overall, in Christchurch, the STAR framework has shown an ability to adapt well to a wide range of situations, and provide safe and effective treatment at all times. It has also reduced clinical burden in the ICU, by lowering the number of measurements and interventions needed to achieve equivalent control to SPRINT, while improving safety.

A key criterion for success in any protocol is the ability to demonstrate high performance and high safety across patient types, time, clinical practice culture and clinical resources. The results of the STAR protocol in Gyula, Hungary, are used to analyze this criterion. The results show that STAR has comprehensively met these criteria, with the BG distributions of the two cohorts being almost identical even though the clinical practices are significantly different.

Thus, this research shows how a model-based and personalized approach to GC can safely improve care and reduce workload across differing clinical practices.

## Abbreviations

STAR: stochastic targeted
GC: glycemic control
BG: blood glucose
APACHE: Acute Physiology and Chronic Health Evaluation
SPRINT: Specialized Relative Insulin and Nutrition Tables
IQR: inter-quartile range
ICU: intensive care unit
SCCM: Society of Critical Care Medicine
ACCP: American College of Chest Physicians
IV: intravenous
CDF: cumulative distribution function
